# Memoirs of West Indian Fevers

**Published:** 1828-01-01

**Authors:** 


*Memoirs of West Indian Fevers; constituting brief Notices regarding the Treatment, Origin, and Nature of the Disease commonly called* YELLOW FEVER. By JAMES WILSON, M. D. of the Royal Navy. Octavo, pp. 217. Burgess and Hill, October, 1827.

ALTHOUGH WAR has long ceased to afford victims for yellow fever, on a large scale; yet, the disease has not altered its character. Whenever the seasons suit, and the European comes within the range of its pestilential breath, this devouring angel proves itself as merciless and destructive as during the disastrous expeditions and campaigns of the revolutionary war. The number of works which issued from the press, in the course of that prolonged struggle, on the subject of yellow fever—the jarring and conflicting testimonies respecting its nature—and the general inefficacy of treatment in the more concentrated forms of the disease, produced a kind of reluctance in the public mind to listen any longer to the declamations of the brawling controversialists—and the consequence was, that we have had but little on the subject, in the English language, for several years past. On the Continent, indeed, a species of desultory warfare still reigns between the contagionists and anti-contagionists; but, in this country, we are heartily sick of such discussions, as the parties have never been able to convert each other to their respective creeds; nor have they succeeded in producing any general conviction in the minds of the public, as to the true state of the questions agitated.

Dr. Wilson, the author of the work under review, although he touches on all the different points of controversy, yet writes like a medical philosopher and a practical man, investigating calmly, and observing accurately, the phenomena of the disease, its real or supposed causes, and the means which have proved efficient or ineffectual in the treatment. On this account, Dr. Wilson is entitled to a candid perusal, although he may be found to have embraced some tenets and views which are hardly tenable in the present state of medical knowledge.

The work is dedicated to the Medical Commissioners of His Majesty's Navy—Drs. Weir and Burnett; and, in his Preface, the author states it to be his chief object to point out the difference between the *inflammatory* and *congestive* modifications of West Indian fever, and to indicate the principles of practice adapted to each. In this place, Dr. W. takes an opportunity of paying a just tribute to the deep thought and talented researches of the late Dr. Jackson, whose writings on fever were never generally appreciated in the manner they deserved, owing, perhaps, to a want of perspicacity—or, at all events, to want of power in conveying his own thoughts to the minds of his readers.

The work contains five memoirs—the first consisting of cases, illustrating the mild inflammatory—violent inflammatory—intense inflammatory—slight congestive—aggravated congestive—and apoplectic congestive, forms of fever. The second memoir is on the *cause* of yellow fever:—the third memoir develops "*new opinions*" as to the cause of fever:—the fourth memoir discusses the question, whether West India fever be or be not, a peculiar disease:—and the last memoir is dedicated to an investigation of the *manner* in which the cause of fever impresses the body.

We shall pass very lightly over the first memoir. A severe visitation of this endemic among the crew of the Rattle-snake, appears to have furnished Dr. Wilson with examples of the six forms or modifications above alluded to, and we believe they are carefully copied from life. The following extract will convey a good idea of the different grades of the inflammatory genus.

"The most constant and prominent symptoms of the inflammatory were, with or without rigor, frequency and strength of pulse, wiry, compressed, or full; a hot non-secreting condition of the skin, particularly at the præcordia and across the forehead; headach, confined generally to the sinciput, with sense of fulness in the eyes and tightness between the temples; jactitation, and constant rolling or otherwise moving of the head; flushing of face, with prominence, wildness, and sometimes inflammation of the eyes; pain in the back and loins, shooting across the anterior parietes of the abdomen, involving the whole contents in tumult; aching in the lower extremities, especially the knee joints, calves of the legs, and tibiæ; sometimes abdominal tension and tenderness in the early stages, sense of emptiness and exhaustion there as the disease proceeded. In the course of the second evening, the symptoms were generally aggravated, and the stomach in many cases became irritable; but this symptom did not occur in the *intense*, and was very tractable in the *mild* species; in the violent, it was exceedingly unmanageable, and often could not be arrested till fatal symptoms came in its train. Delirium was an early symptom in the *violent* and *intense* species, and was always attended with great danger. Costiveness was generally obstinate at the commencement, but was sometimes succeeded by troublesome purging and tenesmus. The tongue varied much in appearance; generally it was white or yellow, loaded and dry, becoming brown or black; urine scanty, high coloured, and attended with dysury in passing; thirst continued and insatiable. The disease generally ran its course within the fourth day. The treatment was briefly as follows.

" In the *mild* species one moderate bleeding, with cathartics and diaphoretics, in most cases, left the patient convalescent in a few days.

" In the *violent* species it was necessary to bleed largely and repeatedly, *viz.* to the amount of sixty, seventy, or eighty ounces in the first twelve hours; and it was gratifying to observe the benefit resulting. For instance, a man complained of excruciating pain in the head, which he moved incessantly; his eyes were wild, inflamed, and impatient of light; the head, according to his own expression, splitting to pieces; the circulation so hurried and tumultuous, that the body was agitated by every pulsation, and the throbbing of the heart visible through the clothes; the skin scorchingly hot and dry; and the entire aspect indicative of great distress. Yet that man, by copious abduction of blood, free catharsis, and a full dose of calomel and opium, was free from complaint on the second day of the disease, and in a few days more capable of returning to his duty. Such was the general plan of treatment in this form of the disease, and such, in a great majority of instances, the result.

" It was far otherwise in the highest grade of inflammatory fever, or that which I have denominated *intense.* There the vascular action was so overwhelming, and the progress to disorganization so rapid, and often so irresistible, that all endeavours to arrest the disease were unavailing. Symptoms could be mitigated, but the character of the disease was seldom changed, or its force broken: within the first twelve hours the patient was delirious, often furiously; at other times it was the delirium of engorgement and oppression. In both cases, the action of the carotids was tremendous; the face red, and frenzied in expression; the eye sometimes clear, quick, and piercing, sometimes dull and darkly inflamed, always indicative of great cerebral derangement. The skin had an intensity of heat scarcely conceivable, particularly on the breast, neck, and head. The tongue was parched, hot, and apparently diminished in size; of various colours, generally brownish. The stomach was retentive, and the bowels costive, but not very obstinately so. This state of inordinate action sometimes continued till near the close of the disease, the patient, in such cases, expiring in a fit of violent convulsion. In other cases, the high excitement ceased suddenly, and, as it were, by a momentary act, and was succeeded by coldness of surface, a state of circulation and respiration scarcely perceptible, calmness of countenance, and perfect quiescence of the body, which soon issued in death without a struggle. The disease was of short duration; and no remission could be observed during its course.

" In such cases evacuation was pushed to the farthest extremity that prudence could sanction. Blood was removed at once till some obvious impression was made on the vascular system or morbid condition, but the latter was seldom effected, and this was repeated as often as the symptoms seemed to require the operation, till some favourable change took place, or such collapse or disorganization came on, as absolutely prohibited its further employment. At the same time such assistance was solicited as purging, blistering, scarifying, sponging, &c. could afford. Yet in many cases all was of no avail. The patient has been reduced by blood-letting, till faintness, vomiting, and cutaneous relaxation came on, and in an hour every symptom had risen to its former intensity. The blood-letting was repeated till the same state was induced, and in another hour every thing was to be done again; and thus many times in succession, till destruction of some organ, generally the brain, ensued. There was a fire, so to speak, kindled in the frame, which nothing could quench. I have heard of men, who boasted that they could cure every case of this disease; that was indeed vain boasting, and calls for no remark; but I have read in books of something very similar in pretension. I have no right to measure any man's success by my own; but I may be allowed to doubt, whether in such cases, and in circumstances like mine, such happy results are likely to happen to others." 11.

The congestive genus is a still more formidable and fatal disease—and that which embraced the greater number of Dr. Wilson's cases.

" A sense of stupor, weight, and oppression, rather than pain in the head; a feeling of helpless debility, affecting the spine, most distressing about the sacrum; a paralytic failure of the lower extremities, with pains in the knees and calves of the legs; a pulse having all degrees of celerity and expansion, but always weak, sinking under the finger without resistance; a state of the skin various and difficult to define, but always deficient in tone, sometimes dry and tense, sometimes greasy, and sometimes drenched in sweat; generally without increase of heat, except at the præcordia, where it was confined and smouldering; a most distressing expression of countenance, deadly pale or livid in colour; a drunken idiotic eye, with dilated pupil and sleepy motion; deafness; desire to be left alone; sighing deep and interrupted; early tendency to coma; tension of the hypochondria; and early irritability of stomach; were the principal symptoms by which this division of the disease was characterised.

" In the lowest grade of this genus, *viz.* the *slight*, these symptoms were not all perceptible, and those that were, had a mild and manageable character. Moderate purging, followed by the use of calomel in five grain doses, two, three, or four times daily, with or without opium and antimony, according to the state of the stomach and skin, soon roused the slumbering energies of the brain, adjusted the balance between the nervous and vascular systems, and restored health.

" In the second or *aggravated* species, more forcible measures and greater perseverance were necessary. I generally bled, as soon as possible, to the extent of ten or twelve ounces at first, for even that quantity induced faintness and vomiting; then administered a purgative medicine; and repeated the blood-letting, or not, according to circumstances. Beyond this I seldom thought it safe to abstract blood; but trusted the remainder of the cure chiefly to calomel. And here I think mercury a remedy of great, of paramount utility. When the patient says that he has no pain; is ill, but cannot tell what is the matter with him; when he turns away with a countenance of helpless despondency from the person addressing him, letting the head drop as in hydrocephalus; when the pupil begins to dilate and hearing to fail; when he shrinks on applying the hand to the epigastrium, and vomits every thing which he swallows, and even in larger quantities; then, in my opinion, nothing can supply the place of calomel. But it must be given largely, and perseveringly. From ten to twenty grains, twice or thrice daily, sometimes with opium, sometimes with antimony, or both, and sometimes alone, I have given in such cases, and, aided by blisters, &c. I have reason to be satisfied with the result. Ptyalism will, in most cases, soon be established; but after all that has been written on the subject, I think I have witnessed the perfect benefit of its operation, without that manifestation. I tried turpentine, but it did not answer my expectations.

"In the highest grade of congestive fever, or that which I designate *apoplectic*, I had to deal with a much more formidable and fatal disease. In the preceding species, there were generally some slight premonitory symptoms; but in this the attack was like the effect of electricity. In an instant its subject was seized with giddiness, dull pain of head, and confusion of ideas; a sense of coldness, weakness, and indescribable uneasiness along the spine; spasmodic pains in the legs, and paralytic incapacity of the lower extremities. He lay as if stunned, and labouring under concussion of the brain, with dilatation of the pupils, and a gloomy, despairing countenance. The pulse was rapid or slow, full or small, but always weak. The skin was cold, generally greasy, or covered with cold liquid sweat, sometimes dry and lifeless. The stomach was sometimes irritable from the beginning, and there was a strange compound sensation of desire for drink and loathing when tasted. From this state the patient, in some instances, never rallied, but sunk down rapidly to dissolution: he became perfectly comatose: the blood seemed to stagnate in the brain and large internal trunks, and, with filmed glassy eye, faltering pulse, and involuntary discharges, he soon expired. In other instances there was reaction, but faint, partial, and irregular: then existence was protracted, and hopes entertained which were seldom realized, profuse hæmorrhage coming on and exhausting life.

"In such cases, medicine, I fear, will never effect much; and, placed as we were, I consider the usual methods of treatment altogether useless. In some instances, where vascular action succeeded the first fearful impression, I tried blood-letting, and am persuaded that it did mischief; whether or not it might be useful if hot baths and powerful frictions were premised, I cannot say; but I have the strongest conviction, that, without some such preparatory process, it will only precipitate the fatal event. I endeavoured, by friction occasionally, blisters, and calomel, to rouse the sensorial and vital powers from their appalling lethargy; but I must admit, that, with thirty-five cases of fever on my hands at a time, and without assistance, these means were not followed up to my satisfaction. That no other method of treatment would have had happier results, I cannot pretend to say; but I should not expect much from any method. Still I would not leave the patient to his fate, in hopeless inactive despondency. With proper 'appliances and means,' I would endeavour to arrest the disease by the following means; baths of high temperature, with strong frictions, electricity, and galvanism, stimuli diffusible and permanent, enemata of turpentine, eau de Cologne, &c." 14.

The foregoing graphic sketches of the two genera and six species of the disease are well deserving of record, though our author acknowledges that these different forms were not always so well defined in character at the bed-side, as he has endeavoured to render them in description. The *species* sometimes ran into each other, and mingled like light and shade—and not the species only, but even the genera, were sometimes so blended as to render it difficult to determine which was the more predominant. Some years of subsequent experience have only tended to confirm Dr. Wilson in the truth and fidelity of his original description, (transmitted at that time to the Navy Medical Board,) though he now believes that it would have been more nosologically scientific to have made but one genus of fever, leaving the inflammatory and congestive forms to constitute species of the same. The mode of nosological arrangement, however, is of such little consequence, that we shall not dwell upon it here. Dr. W. has introduced a running commentary on the foregoing descriptions, suggested by more extended experience, but not materially altering the principles of treatment already laid down. This first memoir terminates with a selection of cases in illustration; but for these we must refer to the work itself.

### II. Cause of Yellow Fever.

We all know the controversies to which this subject has given rise; and we are not inclined to renew these altercations. Dr. Wilson comments very acutely on the several causes assigned by different writers—as insolation, or atmospheric heat—marsh miasmata—contagion—contingent contagion—atmospheric constitution, &c. With none of these is our author satisfied, and least of all with contagion. He does not very peremptorily deny the possibility of *contingent* contagion, though he evidently doubts the soundness of the doctrine. He slurs over the convincing facts presented by the fever in the Bann and at Ascension—because he was not an eye-witness of them, which, we think, is rather an awkward way of getting rid of difficult subjects. The following pretty severe philippic on Sir Gilbert Blane has been called forth by the dogmatic and somewhat contemptuous manner in which that venerable physician impoliticly treated those who differed with him on the subject of contagion.

"Perhaps he may extend to me the contemptuous pity, with which he treats a *certain American journalist*, who *dares not to be convinced*, after having considered the arguments adduced by Sir Gilbert Blane, in proof of the contagious nature of West Indian fever. This does not appear the most likely method of obtaining proselytes to a faith, which Sir Gilbert thinks of such importance to humanity. Should not a reasonable being be reasoned with clearly, forcibly, but patiently? Is the mere word of authority, the oracular declaration of any man, however talented, and however exalted, to *convince* another man, who has the power, and uses the privilege, of thinking for himself? But Sir Gilbert Blane tells us that the controversy is to be decided by facts, not by reasoning. Are the facts then so clear, that he who runs may read? Has no reasonable doubt ever arisen in the minds of rational inquirers respecting the import of facts, which, according to Sir Gilbert Blane, ought to remove all doubts and answer all questions for ever? Were all the men who disbelieved, are all the men who yet doubt or disbelieve, either idiots or knaves? for to this conclusion would the *declamation* of Gir Gilbert Blane lead. To answer such questions would be at once idle and insulting. Men of equal penetration, with better opportunity of judging, and not less anxious to learn the truth, have been led to different conclusions from those drawn by Sir Gilbert Blane; nay, some of them have been *driven* by irresistible evidence, and in opposition to early and long cherished modes of thinking, to draw different conclusions. Was Rush, was Jackson, was Hunter, destitute of common sense or common honesty? Are all the men of the present time, who differ from Sir Gilbert Blane, incapable of *observing facts*, or unwilling to interpret their meaning faithfully. Facts are not always obvious at first sight; they are often encumbered by factitious circumstances, or disguised by false appearances; and ordinary observers do not always find it an extremely easy matter to remove such incumbrances and to strip such disguises: we do not know how Sir Gilbert Blane accomplishes these things so perfectly; we are not told how he has learned, so easily and so unerringly, to *interpret the hand-writing on the wall.* It is strange that the men who have had the best opportunities of *observing these facts*, who have lived long, laboured arduously, and I will add conscientiously, amid the perils and perplexities of West Indian fever, have not seen them in the same or even similar light with Sir Gilbert Blane; for I believe, that, since the time of Dr. Chisholm, no respectable practitioner in the West Indies, who has seen much of the disease, has believed it to be inherently and essentially contagious. And it is passing strange, that Sir Gilbert Blane, who has taken so much pains to teach us the right mode of reasoning on medical matters, should here turn round, and tell us, that all reasoning, in such cases, is not only idle but pernicious! I desire to treat Sir Gilbert Blane with respect, and for his character generally, I do feel respect; but such feelings cannot be forced; and I cannot respect dictatorial intolerance in any man, or any place, more particularly in the exercise of a liberal, but not exact, art; and I cannot admire the temper, which would lead a man, in possession of executive power, *to dismiss from the service of their country such men as could not in conscience subscribe to his articles of professional faith.*" 82.

Dr. Wilson, in this memoir, takes an extensive range over, not only various countries between the Tropics, but various localities in the same countries. This memoir is exceedingly creditable to our author's researches, inquiries, and judgment. Dr. W. dwells a long time on the subject of marsh miasmata, and has unnecessarily accumulated proofs that we cannot clearly trace the cause of the yellow fever of the West Indies to mere exhalations from soils of a *marshy* character. This has long been given up; and the more general opinion is, that the cause of fever is an emanation from the *soil,* whether marshy or not, which gives rise to fevers varying according to the nature of that invisible, and hitherto unascertained agent, or the soil which produces it. Our readers are aware that this is the doctrine which we have uniformly maintained in this Journal, from its commencement. We have often employed the term "febrific miasm," as much freer from objection than *marsh* effluvium, or vegeto-animal miasma. The term malaria is, we think, somewhat objectionable, since it conveys the idea of the cause being essentially connected with the *air,* though reason and observation tell us that the cause springs from the *earth,* and is only suspended in, or wafted about by the air. But it is now time to proceed to the author's "new opinions," regarding the cause of yellow fever.

### THIRD MEMOIR.

Dr. W. observes, that the West India islands, and parts of the adjacent coasts of South and North America, constitute the proper soil, and are the perpetual sources, of West Indian fever. From the mouth of the Amazon to Charlestown in one direction—and from Barbadoes to Tampico in another, the causes of this disease are in constant, though unequal force. It sometimes, as is supposed, extends to New York, and even to the southern parts of Europe. At all events, it reaches from the Equator to the 32d degree of North latitude—and from the 60th to the 98th degree of West longitude. Now, the space included in this vast range exhibits a great variety of aspect, soil, and atmospheric temperature; and, on comparing these with other parts of the world, we can perceive nothing to account for this uniform fertility in the production of a febrific miasm causing yellow fever:—nothing in the heat of the air, the quality of the soil, or the condition of the subject. We must, therefore, after all, take for granted, that there is a *something* generated in the earth, and diffused in the air, to account for this wide spread of fever. Dr. W. comes to the point which we have so long insisted on. "This atmospheric influence, it is reasonable to suppose, is derived from the earth's surface; for we have no notion—can have no notion, of any thing contaminating the air, excepting the earth or its productions."

Dr. W. thinks that this cause, if ever to be discovered at all, will be found by a careful and scientific survey of the more superficial parts of the crust of the globe. We much doubt whether the labours of the geologist and chemist will ever be able to detect the febrific miasm. But, by this, we do not wish to discourage their enquiries. Like the search for hidden treasures, the harvest will reward the toil. This task our author assigns to the medical officers of the army, scattered, as they are, in various parts of the world, and qualified as they are to undertake the investigation. In the mean time, Dr. Wilson himself throws out some hints which he hopes may prove serviceable to those who have better opportunities for prosecuting the inquiry.

He remarks that lime, of secondary formation, is a predominating ingredient in the surface of the West Indies; while, in many places, there is much recent volcanic matter. Where such kind of surface is most conspicuous, from being scantily covered with earth or herbage, *there* the West India fever is peculiarly frequent and severe—"if the more palpable cause of the disease exist in the vicinity." The foregoing position is exemplified by the following curious topographical fact.

"Rock Fort is placed three miles east of Kingston, at the eastern extremity of the plain of Liguanea, where it is narrowed to the breadth of a few yards, being bounded southward by the sea, and northward by a precipitous mountain. The contracted and scanty soil, east and west, is alluvial, but dry, with the exception of a small spot to leeward; and the appearance of the whole would not give an impression of insalubrity, the sea washing one side, the structure beneath being coral, and the mountain, which rises behind a naked calcareous mass, destitute of soil and vegetation. Yet this has been one of the most fatal spots to our troops of any in the West Indies, the detachments employed in its garrison having been swept off in rapid succession by West Indian fever. The officers' quarters stood at a short distance from the general barracks; on a dry indurated foundation, close to the mountain, and occupied presumptively a more healthy site than even the general building. But experience has shown that it was more unhealthy. So unhealthy was it, and so concentrated is the cause of fever upon it, that scarcely an individual quartered there, if lately from Europe, escaped with life. It is for the present abandoned." 128.

Even the naval force suffered severely on this station. A well of excellent water springs on this spot, and there the boats' crews were employed in watering the ships. Entire crews were swept off by the fever, and scarcely any escaped who visited that fatal spot.

Stoney Hill is next adduced. The barracks there stand 1300 feet above the level of the sea, and the air is comparatively cool. There is nothing like a marsh in the vicinity: yet it is extremely unhealthy. Dr. W. informs us, however, that this hill, from its base to its summit, is one great mass of calcareous rock, intersected by deep fissures, and split, in many places, into large fragments, heaped on each other, and crumbling into powder. Over its whole surface, there is scarcely any soil, and little grass, weed, or herbage of any kind. "But it has been, and is still, generally covered with forest trees, the roots of which are seen expanded over, and clinging to, the naked rock, the extreme fibres dipping into the crevices, and hiding themselves amid the *detritus of the rock, decaying leaves, and other ligneous matters which are lodged there.*" We apprehend that Dr. Macculloch, and others of that school, would be at no loss to find a source of malaria, even on the declivities of Stoney Hill.

"Fort Haldane, at Port Maria, occupies the extreme point of a promontory, which projects considerably from the main land, and divides the bay into two basin-like recesses. The promontory, which is 150 feet above the sea, is about 200 feet across, is so nearly perpendicular, and so much alike in its faces, that it has the appearance of an artificial structure reared for the defence of the harbour. It is formed of a pure carbonate of lime, so white and regular as to look like a whitewashed wall; it is level, smooth, and dry on the surface; and when it was chosen as a point of defence for the construction of a fort and barracks, it was probably thought to be one of the most healthy situations in the

West Indies. Merely looking at it as an elevated, regular, dry mass of limestone, washed on three sides by the sea, we should think so still; but the experience of the troops, and the sick returns, prove it to be, in every way, the reverse. So productive of fever and so deadly has it proved, that for some years past it has not been garrisoned. In November, 1824, on urgent representations by the local authorities, that troops were necessary, from apprehended insurrections of the slaves, a detachment of the 50th regiment was sent to occupy the barracks. Fever appeared among them so early, and was so destructive, that, at the expiration of six weeks, the detachment was removed, having lost, I believe, one-third of its number from fever, and the remainder mostly suffering from its effects; and this happened at a season generally healthy, to troops who had been some years in Jamaica, and who were landed in health. Two streams fall into the bay, one on each side of the headland, at about a quarter of a mile distance. *They move slowly, and their banks are covered with mangroves, which, it is to be presumed, furnish the more palpable cause of the fever; but it is remarkable, that the inhabitants of the village of Port Maria, which is situated on both sides of one of the streams, do not appear to suffer from their situation.*" 130.

There are many examples on record, similar to the above. It is by no means impossible, that those who live in the very vicinity of a source of malaria may escape, while those who are elevated above this source, and at some distance, may sustain its most deleterious influence. The current of the prevailing winds, and other circumstances, should here be carefully investigated, as miasmata may be carried towards, or descend, and become *accumulated on*, a lofty eminence, in the neighbourhood of a malarious source, while the same miasmata may never acquire a sufficient concentration in the spots which gave them birth. We believe that this explanation will apply to many unhealthy localities.

Dr. Wilson goes on to give us very interesting sketches of the medical topography of Port Royal—Port of Spain—Fort Louis in the Island of Martinique—Guadaloupe—St. Domingo—Barbadoes, and many other places, shewing how very generally it happens, that high and rocky elevations in the vicinity of mangrove swamps or morasses, are ten times more destructive to life, as the hot-beds of yellow fever, than the valleys or swamps themselves.

The author has been particularly struck with the prevalence of *lime* in all those situations, in the West Indies, which are remarkable for insalubrity, but modestly declines drawing any general conclusion as to the connexion of the one with the other. He, therefore, proceeds to what he calls the more palpable cause of yellow fever.

"This, I believe, is furnished by wood, being a gaseous product of trees and shrubs, in a state of decomposition; generally given out by them in a cut or dried state, but which may arise from a living forest, trees being capable, in different parts of their frame, of simultaneous growth and decay; and further, that wood, after it has passed from the green to the dry state, is still capable of generating the cause, certain degrees of heat and a certain quantity of moisture being supplied. I therefore believe, that decomposing vegetable matter, in a certain sense, but not in the sense generally received, furnishes the cause of West Indian fever; as it is not to herbaceous but *ligneous* matter that I trace it, and on which I shall endeavour to show its dependence." 139.

This, we conceive, is a nice distinction, and, considering that hill-fevers, wood-fevers, jungle-fevers, have been described for 20 or 30 years past, we see no great "novelty" in the opinions here emitted. Dr. Wilson goes on to prove his *ligneous* origin of yellow fever, by shewing that it occurs in ships, where "marsh miasmata are not necessary to its production." Our author does not attempt to explain "what the manner of that process is, and what is the nature of its products;" but only pledges himself to prove that such febrific process does occasionally exist in ships. He objects to the supposition, that a foul hold can be the cause of the sickness—principally, it would appear, from what occurred in the Iphigenia and Rattlesnake, where the yellow fever prevailed extensively, although no foulness could be detected in their holds, and circumstances rendered it highly improbable that the disease could have arisen from terrestrial exhalations. One of the medical gentlemen appointed to examine the above-mentioned ships, and report on the cause of the sickness, was Dr. Bancroft; and it appears that the fever in question completely upset all his former writings, as to the origin of the disease!

"But Dr. Bancroft, whose labours in this field are so well known and so highly appreciated, who has collected so much information and exerted so much talent to prove, that this disease depends on earthy exhalations for its cause, as certainly as does intermittent fever, finds the whole of his elaborate and highly-finished structure sapped by the fever in the Iphigenia. He finds there a stumbling-block, which he had not expected and cannot remove, and he fairly confesses the insuperable nature of the difficulty." 151.

All the usual sources of the febrific miasm having been disproved, Dr. Wilson comes to the conclusion, "that it arises from the decomposition of the wooden materials of the ships themselves, and from such loose timber as they may contain." Dr. Wilson's arguments do not carry conviction to our minds; for we cannot imagine why the same decomposition should not go on in all climates of as high a temperature as the West Indies, which, however, is not the case. We shall offer the following quotation from his arguments.

"The extent, however, to which the process is carried, and the nature of its results, are modified by the previous condition of the wood, the degrees of heat, and, probably, by interior arrangements in individual ships. When a ship recently built arrives at the hottest season of the year, continues for weeks in harbour, and when, as happened in the Rattlesnake, the holds are cleared, and high temperature kept up in them by means of stoves, the process will be rapid and complete. The fever will appear early, and proceed rapidly; but when it ceases, it will cease finally. Men fresh from Europe entered on board such a ship, after the fever has ceased, if they avoid the cause of disease on shore, will continue as secure from West Indian fever as if they served in any other part of the world. On the other hand, when a ship arrives during the cooler months, is kept much at sea, and stoves are not let down into the holds, the process will often be slow and imperfect: in some cases it may never take place. In such ships there will not be severe and sweeping visitations of fever; but then there will never be security. Men will continue to fall under the influence of the disease, till the last day of the ship's term of service on the station. These things are frequently exemplified in different ships in the West Indies." 154.

It is acknowledged by all, that mangrove banks and shores are peculiarly productive of the cause of fever, wherever they are found. The following description of the mangrove production and decay is certainly favourable to the *ligneous* doctrine.

"The mangrove grows and runs to decay rapidly. The branches, after rising a few feet, bend towards the root, on which they engraft themselves; from the bow thus formed other branches spring up, which, in like manner, insert their extremities into different parts of that from which they grow, and so on, till an impenetrable dwarf forest is spread over the surface of the water. While the lower part of this *water forest* is undergoing rapid decomposition, the upper part is in a state of luxuriant growth and beautiful verdure, so that the appearance of the whole is singular and striking: it looks like a piece of basket-work supporting a shrubbery. From its structure and habits, the mangrove appears destined to speedy decay, to which surrounding circumstances are highly favourable. In consequence of the rise and fall of the tides, part of it is alternately wet and dry, and the whole is exposed to the influence of high temperature; hence, whatever deleterious product is furnished by decomposing wood, must be furnished abundantly here." 164.

### MEMOIR THE FOURTH.

We must pass over this memoir, without being able to offer any specimens from the work. The question is, whether the yellow fever of the West Indies is a disease of a peculiar character—one, in fact, *sui generis*, or merely an aggravated form of the usual epidemics and endemics, the bilious remittent and intermittent fevers? Dr. Wilson is clearly of opinion that the latter supposition is false—and that the disease "arises directly and solely from inanimate matter close to the persons whom it affects, and is as incapable of being produced by any other agency as ague or goitre." He cannot, however, subscribe to the doctrine, that it is a mere aggravation of intermittent fever. The author adduces numerous and very powerful arguments in support of his opinions, and these should be consulted by all who are destined for the West Indies.

### FIFTH MEMOIR.

This memoir is on a subject nearly as obscure as the hitherto inscrutable miasma of yellow fever—namely, the *modus operandi* of this cause on the human frame. The difficulty of this investigation may be easily imagined, when we consider that we are called upon to trace the action of a thing *totally unknown* on functions of which we know *exceedingly little*. All we can do, in the present state of things, we believe, is to accurately note the obvious phenomena which follow the application of this incognizable miasma, without attempting much of the rationale of its operation on the living machine.

After re-enumerating the symptoms of the *apoplectic* or congestive species of yellow fever, Dr. W. asks—"is there not here debility in the strictest sense of the word; and is it not positive and direct, rather than secondary and apparent?" He then goes on to observe, that this debility cannot result from any direct agency of the cause of fever on the *vascular* system; but through the medium of the *nervous* system. We agree with Dr. Wilson—and have to remind him that this was the view taken of it by Cullen, and by many writers before Cullen:—it *is* the view of it which is now taken by all the best writers and the most accurate observers. The following passage will convey some idea of Dr. Wilson's views of the *modus agendi* of the febrific miasm.

"It is probable, that in some cases the nervous power is *abstracted*, in others only *obstructed*.

"The first condition is induced, I believe, in pure congestive fever, (for a certain degree of congestion happens in all,) and the extent of congestion is commensurate with the quantity of *abstraction*. In the worst form of congestive fever, it would appear that the nervous power is so completely withdrawn, and receives so little supply, as to be unequal to the carrying on of the functions of life; that it is so subverted, as to be unsusceptible of repair, and that death is the effect of direct exhaustion, and consequent stagnation. In the slighter forms, it appears, though the nervous power is *abstracted* to a certain extent, that sufficient remains to supply sensation, and *something* essential to vital action, till, in favourable circumstances, the loss is repaired, and derangement appears chiefly in the vascular system.

"On the other hand, when the nervous power is only *obstructed* by the febrile cause, the inflammatory form of fever will follow, the force of inflammatory action being proportioned to the extent and duration of the *obstruction.* In this form of the disease, as in the other, the first step of derangement in the vascular system will be congestive, but greatly different in its nature and results. Here, as soon as the cause of *obstruction* is exhausted, or rather perhaps arrested, by some inherent power of the living body, reaction takes place, various in degree in various instances; in some rising to the most intense inflammation, destroying organs essential to life, by a process somewhat like gangrene, in others proceeding, with less violence, to a regular issue in suppuration, and in others affecting chiefly the investments of viscera, and with tendency to terminate in effusions of serum and coagulating lymph." 201.

We do not clearly understand what is meant to be conveyed by the term *abstraction* of the nervous power. The fumes of charcoal—a certain dose of prussic acid—a concussion of the brain—the ablation of a limb by a cannon ball—nay, a powerful mental impression will *arrest* more or less completely the supply of nervous power from the sensorium to the heart and all other organs of the body, occasioning most of the phenomena produced by a great dose of the febrific miasm in a concentrated state, as seen in the apoplectic species of yellow fever. This, we imagine, is as good an illustration of the *modus agendi* of the cause of yellow fever as we can have—and it points out the danger of incautiously abstracting blood in such a condition of the system, as forcibly as any thing can do. The reaction which follows is generally proportionate to the depression that precedes. If reaction do *not* follow at all, Dr. W. seems to call this *abstraction* of the nervous power. If reaction succeed, then it was only *obstruction* of the same. The following passage is interesting, whatever we may think of the explanation which is attempted to be given.

"I have seen a remarkably stout young man, of sanguine temperament, labouring under the first impression of fever, which literally struck him down in an instant. With a rapid, weak, irregular pulse, sunk eye, haggard countenance, and cold skin, he lay prostrate in great distress, but incapable of describing his sensations. There were languor and feebleness, which he tried in vain to resist; confusion of thoughts; tremors, not like the rigor of intermittent fever, but an affection like that which arises from alarm; a feeling of extreme, but unspeakable suffering, extending from the spine to the umbilicus, involving the abdominal contents in tumult; and a state of the skin in which reduction of temperature was not the most striking, though most easily defined, deviaation from that of health. When the hand was applied to the surface, there was communicated a sensation, similar to that which is experienced by touching the scalp, when separated from the subjacent muscles, a sensation of want of vital connexion with the parts beneath. And yet in two hours all those signs of diminished vitality had disappeared, and the most furious inflammatory action followed. The case was a fatal one. Blood-letting and other means of reduction were urged to the uttermost, without making more than momentary impression on the disease. The force of the circulation was for an instant abated, and then rose again to the height of its former violence; and thus, over and over again, till the structure of the brain was subverted.

"Now, in contemplating such a case, we are first of all struck with the two great stages into which it is divided, and then with the necessary connexion between them. In the first we see the nervous power *obstructed*, or suspended in its operation, not arrested in its source or abstracted from the system; and, in the second, we observe, when the suspending or *obstructing* cause is removed, the force accumulated by inaction and continued supply, poured forth with irresistible impetus, and giving rise to such intensity of inflammatory action, as puts to defiance all our means of staying its course. At least, in comparing the two phases of the disease, the subsidence of its first, and the rising of its second stage, the conviction is forced upon us, that they are not fortuitous and inconsequential, but are necessarily connected the one with the other, as cause is with effect; and moreover, that in the origin and progress of this disease, there is something essentially different from what happens in congestive fever.

"*There* we observe, as in the first stage of this, failure of nervous, and feebleness of vascular, power, but of a much deeper and more enduring character. In the inflammatory form of fever, the stage of exhaustion, connected with nervous *obstruction*, is always short, sometimes imperceptible, or rather, in the common routine of complaint and inquiry, it is not perceived. But in the congestive form, arising from the *abstraction* of nervous power, the exhaustion lasts long, in the worst forms, till the last. There is, analogous to what happens in inflammatory fever, failure of nervous power and vital action; but, unlike what happens in that form of the disease, the reparation is slow and scanty, or altogether wanting. In some there is no supply consequent on *abstraction*, in others there is supply, but it is always slow in accession, often inadequate in quantity. It is, when compared with the overflowing energy of inflammatory fever, as a summer brook to the force and celerity of the Ganges." 203.

We have greatly overstepped our intended limits—led on by the interest of the subject, and the talent and observation displayed by the author of the work under review. The minute and accurate details of medical topography—the forcible and faithful delineations of fever in its various forms between the Tropics—the ingenious speculations on its etiology—the rational modes of practice inculcated—and the extremely important suggestions as to prevention, which Dr. Wilson has presented to the public in a small and modest octavo, are so highly creditable to the author, that he has deserved, and will obtain, an elevated niche in the temple of medical fame. Were we destined once more to visit the western Tropics, and obliged to limit our library to *a single volume*, we should have little hesitation in taking with us the five memoirs of Dr. John Wilson.

---

